# The Association between Low Fetal Fraction of Non-Invasive Prenatal Testing and Adverse Pregnancy Outcomes for Placental Compromise

**DOI:** 10.3390/diagnostics14101020

**Published:** 2024-05-15

**Authors:** Soo-Hyun Kim, You-Mi Hong, Ji-Eun Park, Sung-Shin Shim, Hee-Jin Park, Yeon-Kyung Cho, June-Seek Choi, Joong-Sik Shin, Hyun-Mee Ryu, Moon-Young Kim, Dong-Hyun Cha, You-Jung Han

**Affiliations:** 1 Department of Obstetrics and Gynecology, CHA Gangnam Medical Center, CHA University School of Medicine, Seoul 06135, Republic of Korea; soohyunkim@chamc.co.kr (S.-H.K.); hong4136@chamc.co.kr (Y.-M.H.); ogshinyss@chamc.co.kr (S.-S.S.); coolsome72@chamc.co.kr (H.-J.P.); mahler9@chamc.co.kr (Y.-K.C.); juneobgy67@chamc.co.kr (J.-S.C.); shinjs@cha.ac.kr (J.-S.S.); mykimdr410@chamc.co.kr (M.-Y.K.); chadh001@chamc.co.kr (D.-H.C.); 2 Center for Genome Diagnostics, CHA Biotech Inc., Seoul 06125, Republic of Korea; 3 Department of Obstetrics and Gynecology, CHA Bundang Medical Center, CHA University School of Medicine, Seongnam 13497, Republic of Korea; hmryu@cha.ac.kr

**Keywords:** non-invasive prenatal testing, fetal fraction, placental compromise, adverse pregnancy outcomes, hypertensive disease of pregnancy

## Abstract

(1) Background: Non-invasive prenatal testing (NIPT) is a screening test for fetal aneuploidy using cell-free fetal DNA. The fetal fragments (FF) of cell-free DNA (cfDNA) are derived from apoptotic trophoblast of the placenta. The level of fetal cfDNA is known to be influenced by gestational age, multiple pregnancies, maternal weight, and height. (2) Methods: This study is a single-center retrospective observational study which examines the relationship between the fetal fraction (FF) of cell-free DNA in non-invasive prenatal testing (NIPT) and adverse pregnancy outcomes in singleton pregnancies. A total of 1393 samples were collected between 10 weeks and 6 days, and 25 weeks and 3 days of gestation. (3) Results: Hypertensive disease of pregnancy (HDP) occurred more frequently in the low FF group than the normal FF group (5.17% vs. 1.91%, *p* = 0.001). Although the rates of small for gestational age (SGA) and placental abruption did not significantly differ between groups, the composite outcome was significantly higher in the low FF group (7.76% vs. 3.64%, *p* = 0.002). Furthermore, women who later experienced complications such as HDP or gestational diabetes mellitus (GDM) had significantly lower plasma FF levels compared to those without complications (*p* < 0.001). After adjustments, the low FF group exhibited a significantly higher likelihood of placental compromise (adjusted odds ratio: 1.946). (4) Conclusions: Low FF in NIPT during the first and early second trimesters is associated with adverse pregnancy outcomes, particularly HDP, suggesting its potential as a predictive marker for such outcomes.

## 1. Introduction

Noninvasive prenatal testing (NIPT) is a screening test for fetal aneuploidy using cell-free fetal DNA with high sensitivity and specificity [1,2]. In NIPT, cell-free DNA (cfDNA) in maternal plasma is analyzed using high-throughput sequencing methods. During pregnancy, cfDNA contains both maternal and fetal fragments [3]. Maternal fragments of cfDNA are released primarily from the hematopoietic cells [4,5], whereas fetal fragments of cfDNA are derived from apoptotic trophoblast of the placenta [6,7,8].

Fetal fraction (FF) is defined as the percentage of fetal cfDNA to total maternal cfDNA in maternal circulation. The FF measurement is important for controlling the quality and statistical confidence of NIPT results [9,10]. The minimum FF threshold for a reliable test result varies depending on assay technology but is commonly between 2–4%. Low FF is the most common cause of no-call results of NIPT [11], and has also been associated with fetal aneuploidy [1,12,13,14,15]. Some studies have shown an increased risk of fetal aneuploidy in women with low FF, ranging from 2.7% to 23.3% [14,15]. FF is influenced by many biological factors [16,17,18,19,20,21,22,23,24,25,26], and increases with advanced gestational age [16,17] and multiple pregnancies [18,19]. FF decreases with increasing maternal weight, possibly due to increased maternal cfDNA resulting from apoptosis of adipose cells [20,21,22]. It is also affected by other factors such as ethnicity [18], maternal autoimmune disease [23,24], and low molecular heparin treatment [25,26].

Indeed, the origin of fetal cfDNA is not a fetus, but a placenta [27]. For this reason, it has been hypothesized that the concentration of fetal cfDNA reflects placental pathology [27]. FF may serve as an important marker, like traditional maternal serum markers [28,29], for predicting adverse pregnancy outcomes associated with placental dysfunction. Previous studies have shown the association between the level of FF and adverse pregnancy outcomes [30,31,32,33,34,35,36,37]. However, the results were inconsistent. Recent data have demonstrated an association between low FF and adverse pregnancy outcomes [33,34,35,36,37].

The objective of this study was to evaluate the correlation between the FF level in NIPT and adverse pregnancy outcomes. Additionally, we sought to investigate whether a low FF is associated with adverse pregnancy outcomes related to placental compromise.

## 2. Materials and Methods

### 2.1. Study Design

This was a retrospective observational study of 1393 women with single pregnancies who underwent NIPT and gave birth at CHA Gangnam Medical Center from July 2019 to June 2021. This study received approval from the ethics committee of CHA Gangnam Medical Center (IRB number: 2023-11-012-002). The inclusion criteria were as follows: 1. age at least 20 years old; 2. singleton gestation; 3. women who underwent NIPT and delivered at CHA Gangnam Medical Center during the above period. Women were excluded if they had multiple pregnancies, positive NIPT results, subsequent abortion, subsequent intrauterine fetal death, or a fetus with major congenital malformations.

### 2.2. Data Collection

All NIPT was performed using next-generation sequencing at CHA BiotechInc. with Ion GeneStudio S5 system (ThermoFisher Scientific, Seoul, Republic of Korea). Electronic medical records of patients were reviewed for data collection including basic maternal characteristics such as maternal age, BMI, gestational age at the time of NIPT, presence of IVF conception, gestational age at delivery, and mode of delivery. The medical information along with indicators suggestive of potential placental compromise, such as hypertensive disease of pregnancy (HDP), placental abruption, and small for gestational age (SGA), were also collected. FF values defined as the percentage of fetal cfDNA to total maternal cfDNA in maternal circulation were recorded from the hospital’s prenatal screening database when they underwent NIPT. 

### 2.3. Outcome Measurements

The primary outcome was defined as both a composite outcome and each adverse pregnancy outcome representing placental compromise, including HDP, placental abruption, and SGA. The definition of HDP was those from the international society for the study of hypertension in pregnancy. Hypertension in pregnancy continues to be defined as a clinic systolic blood pressure ≥ 140 mmHg and/or a diastolic blood pressure ≥ 90 mmHg. We included all types of hypertensive disorders, chronic hypertension, essential hypertension, secondary hypertension, masked hypertension, transient gestational hypertension, pre-eclampsia, and superimposed chronic hypertension. Placental abruption was defined as the separation of the placenta before or during labor. We categorized neonates who were in the <10th percentile of birth weight for their gestational age as SGA, based on a national reference [38]. The secondary outcomes included gestational diabetes mellitus (GDM) and preterm delivery (PTD). According to the United States Preventive Services Task Force (USPSTF) recommendations, diagnosis of GDM depended on a 50 g oral glucose tolerance test at 24–28 weeks followed by 100 g oral glucose tolerance test. We conducted the 100 g oral glucose tolerance test if the result of the 50 g oral glucose tolerance test was 140 mg/dL or higher. For the 100 g oral glucose tolerance test, diagnosis was based on meeting two or more of the following criteria: fasting blood sugar levels exceeding 95 mg/dL, blood sugar levels exceeding 180 mg/dL after one hour, blood sugar levels exceeding 155 mg/dL after two hours, or blood sugar levels exceeding 140 mg/dL after three hours. Based on the ACOG definition, PTD is classified using gestational age at delivery as follows: total PTD is defined as birth before 37 weeks of gestation, early PTD refers to birth before 34 weeks of gestation, and late PTD refers to birth between 34 and 36 weeks of gestation. Additionally, we classified women who did not develop any pregnancy-related complication, including HDP, placental abruption, SGA, GDM, and PTD, as non-pregnancy complication (NPC). The covariates considered in this study were maternal age, BMI, delivery mode (classified as vaginal delivery and cesarean section), gestational age on NIPT, gestational age at delivery, and method of conception (divided into in vitro fertilization (IVF) and others). BMI was classified as obesity according to the standards of the Korean Society for the Study of Obesity (KSSO), with 25 or higher being defined as obesity. Advanced maternal age was defined as women who are 35 years or older according to the definition by the American College of Obstetricians and Gynecologists (ACOG).

### 2.4. Statistical Analysis

In this study, categorical variables, continuous variables with normally and non-normally distribution, were expressed as *N* (%), mean standard deviation (SD), and median, respectively. Kruskal Wallis test, ANOVA test, Chi-square test, or Fisher’s exact test were used to analyze continuous and categorical variables, respectively. Spearman correlation analysis was applied to examine the relationships between plasma FF and maternal characteristics. Multiple linear regression analysis was also used to evaluate the relationship between the level of FF and gestational age, BMI, and maternal age. In addition, logistic regression analysis was performed to assess the association between low FF and adverse pregnancy outcomes. The correlations are expressed as an odds ratio with a 95% confidence interval (CI), with a value greater than one indicating increased odds. Adjusted covariates included maternal age, gestational age, and BMI on NIPT, IVF, or not. We divided the FF value into quartiles based on the 10th, 25th, 50th, 75th, and 90th percentiles, defining values less than the 25th percentile as low FF and those that exceed the 75th percentile as high FF. The data analyses were performed using Statistical Package for Social Sciences version 26.0 (IBM Corp., Chicago, IL, USA). *p* < 0.05 denoted statistical significance.

## 3. Results

### 3.1. Characteristics of Study Population

Among the 2373 women that underwent NIPT during the study period, 1397 women delivered at this hospital. Seventeen showed positive results in NIPT, six were excluded due to loss of follow-up, and eight were excluded due to major congenital malformations. Three women delivered and were included in this study. A total of 1393 women were analyzed. The low FF group consisted of 348 women, representing 25.0% of all women, while the normal FF group consisted of 1045 women, accounting for 75.0%. The demographic characteristics of the study population, including maternal age, BMI, gestational age on NIPT, gestational age at delivery, IVF conception, and delivery mode, are shown in Table 1. There were significant differences in maternal age, BMI, IVF conception, and delivery mode between the low FF group and the normal FF group. The proportion of women with a BMI on NIPT ≥ 25, those who conceived through IVF, and those who underwent Cesarean section were statistically significantly higher in the low FF group compared to the normal FF group (27.0% vs. 8.6%, *p* = 0.001, 32.5% vs. 24.8%, *p* = 0.008, and 76.6% vs. 69.4%, *p* = 0.014, respectively). The maternal median age was 37 years (minimum: 23 years old; maximum: 45 years old). The maternal median gestational age on NIPT was 12.1 weeks (minimum: 10.9 weeks; maximum: 25.4 weeks). The proportion of women with maternal age on NIPT ≥35 was statistically significantly higher in the low FF group compared to the normal FF group (76.6% vs. 74.2%, *p* = 0.014). There was no statistically significant difference in gestational age on NIPT between the two groups, with median 12.25 weeks for the low FF group and median 12.27 weeks for the normal FF group. None of the women who had heparin therapy or hemoglobin-related hemoglobinopathies were Korean.

### 3.2. Plasma FF and Pregnancy Outcomes

The median of FF was 8.83% with a range from 1.77% to 24.16%. The average FF was 9.45%. The cut-off for FF at the 25th and 75th percentile was 7.12% and 11.22%, respectively. Using multiple linear regression analysis, we analyzed the relationship between fetal fraction and gestational age, maternal age, and BMI. The R-squared was 0.092, the adjusted R-squared was 0.090, and the F-value was 46.78, with a *p*-value of <0.001. Although the explanatory power is low, there was evidence of a linear relationship.

The women having HDP, placental abruption, SGA, GDM, and PTD in the study population were 38 (2.73%), 5 (0.36%), 25 (1.79%), 77 (5.53%), and 81 (5.81%), respectively. A total of 65 women (4.67%) experienced composite outcomes, including HDP, SGA, and placental abruption. Among the 81 women with PTD, 19 (23.5%) had preterm deliveries early PTD (<34 weeks), while 62 (76.5%) had deliveries late PTD (34–36 weeks). Plasma FF was negatively correlated with maternal age, weight, and BMI. Table 2 presented the distribution of pregnancy outcomes between the low FF group and the normal FF group. The incidence of HDP is significantly increased in the low FF than the normal group (5.17% vs. 1.91%, *p* = 0.001). The incidence of SGA and placental abruption were not significantly different between the two groups. The composite outcome was significantly higher in the low FF group than the normal group (7.76% vs. 3.64%, *p* = 0.002).

We compared the high FF group, defined as FF exceeding the 75th percentile, with the low FF and normal FF groups, but found no statistically significant differences in the incidences of HDP, composite outcomes, SGA, and placental abruption.

The distribution of plasma FF on NIPT based on subsequent pregnancy-related complications is demonstrated in Table 3. Among 1393 women, non-pregnancy complication (NPC) women accounted for 86.5% (1205 women). According to the plasma FF levels on NIPT, throughout the entire range from the 10th percentile to the 90th percentile, the FF values of the non-pregnancy complication (NPC) women were consistently higher than those any experienced complications such as GDM, HDP, PTD, and composite outcomes. Plasma FF levels on NIPT were found to be especially lower in women who later experienced complications of HDP, GDM, or composite outcomes compared to women who did not develop any pregnancy-related complications (NPC) (*p* < 0.001). There was no significant difference in FF between women who developed PTD and women with NPC. In addition, the result of the correlation analysis between plasma FF and maternal characteristics is presented in Table 4. Of covariates, age, height, and BMI exhibited statistically significant negative correlations with FF on NIPT (r = −0.074, −0.284 and −0.282, respectively), with weight demonstrating the strongest correlation coefficient. Height and gestational age on NIPT did not show a statistically significant correlation with FF on NIPT.

The association between FF on NIPT and pregnancy outcomes in crude and adjusted models was presented in Table 5. Adverse pregnancy outcomes, including composite outcomes, HDP, PTD, and GDM, showed statistically significant associations with low FF of NIPT in crude model (OR 2.229, 95% CI 1.340–3.708, *p* = 0.002, OR 2.795, 95% CI 1.461–5.348, *p* = 0.002, OR 1.638 95% CI 1.019–2.633, *p* = 0.042, and OR 2.003, 95% CI 1.246–3.221, *p* = 0.004, respectively). However, after adjusting for maternal age, BMI, and IVF status, only the composite outcome and HDP remained statistically significantly associated. Among individual components, HDP showed the highest adjusted odds ratio, followed by the composite outcome. Low FF was significantly associated with an increased risk of HDP (adjusted OR 2.058, 95% CI 1.018–4.161, *p* = 0.044). The low FF group was found to be significantly more likely to develop placental compromise with an adjusted odds ratio of 1.946 (95% CI 1.130–3.351, *p* = 0.016). However, there was no significant association between low FF and SGA, and placental abruption. Although secondary outcomes such as GDM and PTD appeared to show an increased risk in the low FF group, the adjusted risk was not statistically significant.

## 4. Discussion

This study demonstrates that low FF of NIPT in the first and early second trimesters is associated with adverse pregnancy outcomes for placental compromise. Placental compromise may be explained by abnormal placentation in early pregnancy [39]. Shallow placentation could lead to poor access to maternal blood supply and decrease fetal cfDNA. This hypothesis was supported by [37,40]. The study by Rolnik et al. demonstrated that FF is positively associated with pregnancy-associated plasma protein-A (PAPP-A) and placental growth factor, and suggested that lower FF may be a consequence of smaller placental mass and an early sign of placental dysfunction [40]. In contrast to recent results, previous studies have demonstrated an association between high FF and adverse pregnancy outcomes [30,31,32]. Bauer et al. reported that a significant increase of fetal cfDNA concentrations in the maternal plasma during the second trimester would be a beneficial screening marker for the development of complications in pregnancy [32,41]. Such studies were performed in the second and third trimesters, and the timing of sampling was different from recent data. However, these results have been inconsistent, with some studies indicating no association between elevated FF levels and pregnancy complications [42,43]. There is currently no validated clinical test using absolute placental cfDNA levels or FF to predict pregnancy outcomes. In our study, when comparing the group with a fetal fraction above the 75th percentile to the group with a fetal fraction between 25–75th percentile, there was no statistically significant difference in pregnancy outcomes. The discrepant results with previous studies might be explained by the following two mechanisms. First, the initial failed trophoblast invasion might decrease FF in the first trimester and the subsequent oxidative stress might lead to increased trophoblast apoptosis and FF later in pregnancy with adverse outcomes [27]. Second, the clearance of cfDNA from the maternal circulation might be reduced due to organ dysfunction late in the pregnancy with adverse outcomes [44]. We collected samples in the first and early second trimester and identified the strongest relationship between low FF and HDP as a composite outcome indicating placental compromise. Low FF in early pregnancy has been associated with an increased risk of developing HDP in many papers [33,35,36,37]. In a recent systematic review on the association between low FF and adverse pregnancy outcomes, four out of five studies confirmed a significant correlation between low FF and HDP [45]. In research, like our study with a low FF cutoff as less than the 25th percentile, Gerson et al. reported a higher frequency of HDP in the low FF group (20% vs. 10%; *p* < 0.001) [35] and Yuan et al. observed an increased frequency of pre-eclampsia in the low FF group (OR 2.16, *p* = 0.009) [37]. In studies analyzing the frequency based on the timing of pre-eclampsia onset, inconsistent results were not reported. Rolnik et al. found that in early-onset pre-eclampsia, the fetal fraction is reduced, but such differences were not observed after adjustment for maternal characteristics [43]. However, another retrospective cohort study reported that the lower the FF, the risks for pre-eclampsia <34 weeks and 37 weeks were increased (*p* < 0.001 for all) [40]. Becking et al. demonstrated significantly higher rates of pregnancy-induced hypertension (11.2% vs. 5.3%; *p* < 0.001) and pre-eclampsia ≥34 weeks of gestation (3.7% vs. 1.9%; *p* < 0.005) in the low FF group, but not for pre-eclampsia <34 weeks [46].

According to one systematic review, a negative correlation between maternal age and BMI with fetal fraction was found [47]. Furthermore, LDL, cholesterol, triglyceride level, metformin, heparin and enoxaparin therapy, hemoglobin-related hemoglobinopathies, and physical activity showed negative associations. In our study, maternal age, weight, and BMI were negatively correlated with the fetal fraction (Table 4). The inverse relationship between FF and maternal BMI is well recognized [36,37] and our study also showed consistent results. Among our patient, there is no one who had heparin therapy or hemoglobin-related hemoglobinopathies. Therefore, we could not evaluate the effect of these factors. Our patients were all Korean, meaning that we could not evaluate the effect of ethnicity. As with previous studies, positive correlation was observed between gestational age and the level of fetal fraction of cfDNA in our study.

Although not statistically significant, we observed a trend of negative correlation between the level of FF and assisted reproductive technology conception. Previous studies have reported a negative correlation between them, similar to our study [17,48], and explained that the effect of hormone treatment or impaired placentation in IVF conception may decrease the production of fetal cfDNA [48]. Another possible mechanism is the increase of maternal cfDNA due to increased inflammation and endothelial damage in women with IVF conception [49]. However, an association of low FF with infertility itself cannot be yet explained.

Our result did not show an association between low FF and SGA (birthweight <10th birthweight). This was the same result in other studies [33,36,37,46]. However, Clapp et al. found an increased rate of birthweight ≤5th percentile and ≤10th percentile in women with low FF [34]. In a case-control study, the FF of low-risk pregnancies was lower in developed early-onset, but not late-onset fetal growth restriction [50].

Previous studies have reported increased levels of cfDNA in pregnant women with PTD, suggesting that elevated fetal cfDNA (cffDNA) may be pro-inflammatory and stimulate parturition [32,51]. However, these studies were sampled during the second trimester, differing from our research in the timing of sample collection. Many recent studies have reported no association between PTD and FF [33,35]. Quezada et al. concluded that the measurement of FF at 11–13 weeks’ gestation is not predictive of spontaneous PTD (<34 weeks, 34–37 weeks, <37 weeks of gestation) [52]. 

Although there was a higher frequency of PTD (<37 weeks of gestation) in the low FF group, it did not show statistical significance. We also investigated whether there was an association between low FF and early PTD (<34 weeks) and late PTD (34–37 weeks), respectively, but found no significant correlation. In a recent large-scale cohort study, FF on NIPT at the first and second trimesters was not associated with spontaneous PTD [53]. However, several previous studies demonstrated this association [36,37]. Yuan et al. found an increased risk of PTD <34 weeks of gestation in women with FF <10th percentile [37]. It seems that the cause of different results lies in the diversity of factors contributing to PTD. Spontaneous PTD is a syndrome with multiple etiological factors, and various pathological causes may result in different patterns at the cffDNA level.

Previous studies have reported inconsistent results regarding between low FF and GDM [33,36,37]. Chan et al. reported an increased rate of GDM in low FF, but this study was not adjusted for BMI [33]. Since increased BMI acts as a confounding factor for the development of GDM [20,21,22], it could have influenced the analysis results. Other studies did not find an association between low FF and GDM after adjusting for BMI [36,37]. Initially, in the unadjusted risk estimates, we observed an increased risk of GDM in the low FF group. However, after adjusting maternal age, BMI, and IVF, the risk did not increase significantly. 

Previous studies were conducted during the second and third trimesters, demonstrating an association between high FF and adverse pregnancy outcomes. However, our study was conducted during the first and early second trimesters, and we found that low FF in NIPT is associated with adverse pregnancy outcomes due to placental compromise. Our study had several strengths. First, all NIPT was performed by a single genetic laboratory using the same platform and settings at the first and second trimesters. Second, this study was conducted in a single institution and the same criteria were applied to diagnose adverse pregnancy outcomes. Third, potential confounders such as maternal age, BMI, and IVF were adjusted for analysis. A limitation of this study was the fact that we do not have sufficient power to detect uncommon outcomes. We were also unable to analyze the onset timing and severity of the disease separately. 

## 5. Conclusions

Low FF of NIPT at the first and second trimesters is associated with adverse pregnancy outcomes for placental compromise, specifically with HDP. Since NIPT is mostly performed in the first trimester, it is expected that FF can be used as an important marker for placental compromise such as PAPP-A. Furthermore, we anticipate that by adding FF to previously known multivariate variables, the adverse pregnancy outcomes could be better predicted at an early gestation age. Future research is needed to accurately provide the cutoff values of FF as a prediction marker for adverse pregnancy outcomes.

## Figures and Tables

**Table 1:** Demographic characteristics of study population stratified by fetal fraction.

	Low FF (*n* = 348)	Normal FF (*n* = 1045)	*p*
Maternal age on NIPT (years) *	36.86 ± 3.09	36.30 ± 3.41	0.007
<35	78 (22.4%)	269 (25.7%)	
≥35	270 (77.6%)	776 (74.2%)	
BMI on NIPT (kg/m^2^) **	23.33 ± 3.83	21.22 ± 2.58	<0.001
<25	254 (73.0%)	955 (91.4%)	
≥25	94 (27.0%)	38 (8.6%)	
Gestational age on NIPT	12.25 ± 1.08 (11–18)	12.27 ± 1.26 (11–26)	0.822
Gestational age at delivery	38.5 ± 1.80	38.7 ± 1.46	0.090
IVF conception *	113 (32.5%)	260 (24.8%)	0.008
Delivery mode			
Vaginal delivery *	83 (23.9%)	319 (30.5%)	0.014
Cesarean section	265 (76.6%)	726 (69.4%)	

* *p* < 0.05, ** *p* < 0.005. FF, fetal fraction; BMI, body mass index; NIPT, non-invasive prenatal testing; IVF, in vitro fertilization.

**Table 2:** Pregnancy outcomes stratified by fetal fraction.

	Low FF (*n* = 348)	Normal FF (*n* = 1045)	*p*
Primary outcomes			
Composite outcomes **	27 (7.76%)	38 (3.64%)	0.002
HDP **	18 (5.17%)	20 (1.91%)	0.001
Placental abruption	3 (0.86%)	2 (0.19%)	0.103
SGA	6 (1.72%)	19 (1.81%)	0.909
Secondary outcomes			
GDM **	30 (8.62%)	47 (4.49%)	0.004
PTD *	28 (8.05%)	53 (5.07%)	0.040
<34 weeks	7 (2.01%)	12 (1.15%)	0.211
34–36 weeks	21 (6.03%)	41 (3.92%)	0.091

* *p* < 0.05, ** *p* < 0.005. FF, fetal fraction; HDP, hypertensive disease of pregnancy; SGA, small for gestational age; GDM, gestational diabetes mellitus; PTD, preterm delivery.

**Table 3:** Median and percentiles of FF on NIPT based on subsequent pregnancy-related complications.

	*N*	10th Percentile	25th Percentile	Median	75th Percentile	90th Percentile
NPC	1205	5.80	7.30	8.97	11.37	14.09
GDM *	77	4.23	6.35	8.14	9.80	13.18
HDP **	38	4.76	5.78	7.28	8.33	9.88
PTD	81	4.77	6.13	7.97	11.17	15.15
Composite outcome **	65	4.77	6.08	7.46	8.45	10.67

* *p* < 0.05, ** *p* < 0.005. NPC, non-pregnancy complications; GDM, gestational diabetes mellitus; HDP, hypertensive disease of pregnancy; PTD, preterm delivery.

**Table 4:** Spearman’s correlation between FF on NIPT and maternal characteristics.

	r	*p*
Age (year) *	−0.074	0.005
Height (meter)	−0.015	0.572
Weight (kg) **	−0.284	<0.001
BMI (kg/m^2^) **	−0.282	<0.001
Gestational age on NIPT (day)	0.008	0.759
In vitro fertilization	−0.050	0.064

* *p* <0.05, ** *p* < 0.005.

**Table 5:** Association between FF on NIPT and birth outcomes in crude and adjusted models.

Outcome	Group	Crude			Adjusted *		
		OR	95% CI	*p*	OR	95% CI	*p*
Composite outcome	Low FF	2.229	1.340–3.708	0.002	1.946	1.130–3.351	0.016
	Normal	1.000			1.000		
HDP	Low FF	2.795	1.461–5.348	0.002	2.058	1.018–4.161	0.044
	Normal	1.000			1.000		
SGA	Low FF	0.947	0.375–2.391	0.909	1.128	0.431–2.950	0.807
	Normal	1.000			1.000		
Placental abruption	Low FF	4.535	0.755–27.251	0.098	4.414	0.657–29.671	0.127
	Normal	1.000			1.000		
PTD	Low FF	1.638	1.019–2.633	0.042	1.539	0.931–2.543	0.093
	Normal	1.000			1.000		
GDM	Low FF	2.003	1.246–3.221	0.004	1.163	0.685–1.975	0.576
	Normal	1.000			1.000		

* Adjusted for maternal age, BMI, IVF. HDP, hypertensive disease of pregnancy; SGA, small for gestational age; PTD, preterm delivery; GDM, gestational diabetes mellitus.

## Data Availability

The detailed data presented in this study are available from the corresponding author upon request.

## Abbreviations

The following abbreviations are used in this manuscript:

FF	fetal fraction
NIPT	non-invasive prenatal testing
HDP	hypertensive disease of pregnancy
SGA	small for gestational age
GDM	gestational diabetes mellitus
cfDNA	cell-free DNA
PE	pre-eclampsia
PTD	preterm delivery
USPSTF	United States Preventive Services Task Force

## References

[1] Norton M.E., Jacobsson B., Swamy G.K., Laurent L.C., Ranzini A.C., Brar H., Tomlinson M.W., Pereira L., Spitz J.L., Hollemon D. (2015). Cell-free DNA analysis for noninvasive examination of trisomy. N. Engl. J. Med..

[2] Chen Y.P., He Z.Q., Shi Y., Zhou Q., Cai Z.M., Yu B., Wang T. (2018). Not all chromosome aberrations can be detected by NIPT in women at advanced maternal age: A multicenter retrospective study. Clin. Chim. Acta.

[3] Lo Y.M., Corbetta N., Chamberlain P.F., Rai V., Sargent I.L., Redman C.W., Wainscoat J.S. (1997). Presence of fetal DNA in maternal plasma and serum. Lancet.

[4] Lui Y.Y., Chik K.W., Chiu R.W., Ho C.Y., Lam C.W., Lo Y.M. (2002). Predominant hematopoietic origin of cell-free DNA in plasma and serum after sex mismatched bone marrow transplantation. Clin. Chem..

[5] Snyder M.W., Kircher M., Hill A.J., Daza R.M., Shendure J. (2016). Cell-free DNA comprises an in vivo nucleosome footprint that informs its tissues of origin. Cell.

[6] Flori E., Doray B., Gautie E., Kohler M., Ernault P., Flori J., Costa J.M. (2004). Circulating cell-free fetal DNA in maternal serum appears to originate from cyto- and syncytiotrophoblastic cells. Case report. Hum. Reprod..

[7] Alberry M., Maddocks D., Jones M., Abdel Hadi M., Abdel-Fattah S., Avent N., Soothill P.W. (2007). Free fetal DNA in maternal plasma in anembryonic pregnancies: Confirmation that the origin is the trophoblast. Prenat. Diagn..

[8] Faas B.H., de Ligt J., Janssen I., Eggink A.J., Wijnberger L.D., van Vugt J.M., Vissers L., van Kessel A.G. (2012). Non-invasive prenatal diagnosis of fetal aneuploidies using massively parallel sequencing-by ligation and evidence that cell-free fetal DNA in the maternal plasma originates from cytotrophoblastic cells. Expert Opin. Biol. Ther..

[9] Canick J.A., Palomaki G.E., Kloza E.M., Lambert-Messerlian G.M., Haddow J.E. (2013). The impact of maternal plasma DNA fetal fraction on next generation sequencing tests for common fetal aneuploidies. Prenat. Diagn..

[10] Takoudes T., Hamar B. (2015). Performance of non-invasive prenatal testing when fetal cell-free DNA is absent. Ultrasound Obstet. Gynecol..

[11] Gil M.M., Accurti V., Santacruz B., Plana M.N., Nicolaides K.H. (2017). Analysis of cell-free DNA in maternal blood in screening for aneuploidies: Updated meta-analysis. Ultrasound Obstet. Gynecol..

[12] Rava R.P., Srinivasan A., Sehnert A.J., Bianchi D.W. (2014). Circulating fetal cell free DNA fractions differ in autosomal aneuploidies and monosomy X. Clin. Chem..

[13] Revello R., Sarno L., Ispas A., Akolekar R., Nicolaides K.H. (2016). Screening for trisomies by cell-free DNA testing of maternal blood: Consequences of a failed result. Ultrasound Obstet. Gynecol..

[14] Zhou L., Gao Y., Yuan Y., Guo Y., Zhou L., Liao K., Wang J., Du B., Hou Y., Chen Z. (2015). Effects of maternal and fetal characteristics on cell-free fetal DNA fraction in maternal plasma. Reprod. Sci..

[15] Palomaki G.E., Kloza E.M., Lambert-Messerlian G.M., van den Boom D., Ehrich M., Deciu C., Bombard A.T., Haddow J.E. (2015). Circulating cell free DNA testing: Are some test failures informative?. Prenat. Diagn..

[16] Kinnings S.L., Geis J.A., Almasri E., Wang H., Guan X., McCullough R.M., Bombard A.T., Saldivar J.S., Oeth P., Deciu C. (2015). Factors affecting levels of circulating cell-free fetal DNA in maternal plasma and their implications for noninvasive prenatal testing. Prenat. Diagn..

[17] Ashoor G., Syngelaki A., Poon L.C., Rezende J.C., Nicolaides K.H. (2013). Fetal fraction in maternal plasma cell-free DNA at 11-13 weeks’ gestation: Relation to maternal and fetal characteristics. Ultrasound Obstet. Gynecol..

[18] Hedriana H., Martin K., Saltzman D., Billings P., Demko Z., Benn P. (2019). Cell Free DNA fetal fraction in twin gestations in single nucleotide polymorphism-based non-invasive prenatal screening. Prenat. Diagn..

[19] Galeva S., Gil M.M., Konstantinidou L., Akolekar R., Nicolaides K.H. (2019). First trimester screening for trisomies by cfDNA testing of maternal blood in singleton and twin pregnancies: Factors affecting test failure. Ultrasound Obstet. Gynecol..

[20] Hui L., Bianchi D.W. (2020). Fetal fraction and noninvasive prenatal testing: What clinicians need to know. Prenat. Diagn..

[21] Haghiac M., Vora N.L., Basu S., Johnson K.L., Presley L., Bianchi D.W., de Mouzon S.H. (2012). Increased death of adipose cells, a path to release cell-free DNA into systemic circulation of obese women. Obesity.

[22] Juul L.A., Hartwig T.S., Ambye L., Sørensen S., Jørgensen F.S. (2020). Noninvasive prenatal testing and maternal obesity: A review. Acta Obstet. Gynecol. Scand..

[23] Hui L., Bethune M., Weeks A., Kelley J., Hayes L. (2014). Repeated failed noninvasive prenatal testing owing to low cell-free fetal DNA fraction and increased variance in a woman with severe autoimmune disease. Ultrasound Obstet. Gynecol..

[24] Hui C.Y., Tan W.C., Tan E.L., Tan L.K. (2016). Repeated failed non-invasive prenatal testing in a woman with immune thrombocytopenia and antiphospholipid syndrome: Lessons learnt. BMJ Case Rep..

[25] Burns W., Koelper N., Barberio A., Kelly M.D., Mennuti M., Sammel M.D., Dugoff L. (2017). The association between anticoagulation therapy, maternal characteristics, and a failed cfDNA test due to a low fetal fraction. Prenat. Diagn..

[26] Ma G., Wu W., Lee M., Lin Y., Chen M. (2018). Low-molecular-weight heparin associated with reduced fetal fraction and subsequent false-negative cell-free DNA test result for trisomy 21. Ultrasound Obstet. Gynecol..

[27] Taglauer E.S., Wilkins-Haug L., Bianchi D.W. (2014). Cell-free fetal DNA in the maternal circulation as an indication of placental health and disease. Placenta.

[28] Yuan X.S., Long W., Liu J.B., Zhang B., Zhou W.B., Jiang J., Yu B., Wang H.Y. (2019). Associations of serum markers screening for Down’s syndrome with pregnancy outcomes: A Chinese retrospective cohort study. Clin. Chim. Acta.

[29] Cohen J.L., Smilen K.E., Bianco A.T., Moshier E.L., Ferrara L.A., Stone J.L. (2014). Predictive value of combined serum biomarkers for adverse pregnancy outcomes. Eur. J. Obstet. Gynecol. Reprod. Biol..

[30] Al Nakib M., Desbriere R., Bonello N., Bretelle F., Boubli L., Gabert J., Levy-Mozziconacci A. (2009). Total and fetal cell-free DNA analysis in maternal blood as markers of placental insufficiency in intrauterine growth restriction. Fetal Diagn. Ther..

[31] Farina A., LeShane E.S., Romero R., Gomez R., Chaiworapongsa T., Rizzo N., Bianchi D.W. (2005). High levels of fetal cell-free DNA in maternal serum: A risk factor for spontaneous preterm delivery. Am. J. Obstet. Gynecol..

[32] Dugoff L., Barberio A., Whittaker P.G., Schwartz N., Sehdev H., Bastek J.A. (2016). Cell free DNA fetal fraction and preterm birth. Am. J. Obstet. Gynecol..

[33] Chan N., Smet M.E., Sandow R., Silva Costa F., McLennan A. (2018). Implications of failure to achieve a result from prenatal maternal serum cell-free DNA testing: A historical cohort study. BJOG.

[34] Clapp M.A., Berry M., Shook L.L., Roberts P.S., Goldfarb I.T., Bernstein S.N. (2020). Low fetal fraction and birth weight in women with negative first-trimester cell-free DNA screening. Am. J. Perinat..

[35] Gerson K.D., Truong S., Haviland M.J., O’Brien B.M., Hacker M.R., Spiel M.H. (2019). Low fetal fraction of cell-free DNA predicts placental dysfunction and hypertensive disease in pregnancy. Pregnancy Hypertens..

[36] Krishna I., Badell M., Loucks T.L., Lindsay M., Samuel A. (2016). Adverse perinatal outcomes are more frequent in pregnancies with a low fetal fraction result on noninvasive prenatal testing. Prenat. Diagn..

[37] Yuan X., Zhou L., Zhang B., Wang H., Yu B., Xu J. (2020). Association between low fetal fraction of cell free DNA at the early second-trimester and adverse pregnancy outcomes. Pregnancy Hypertens..

[38] Alexander G.R., Himes J.H., Kaufman R.B., Mor J., Kogan M. (1996). A United States national reference for fetal growth. Obstet. Gynecol..

[39] Lyall F., Bulmer J.N., Duffie E., Cousins F., Theriault A., Robson S.C. (2001). Human trophoblast invasion and spiral artery transformation: The role of PECAM-1 in normal pregnancy, preeclampsia, and fetal growth restriction. Am. J. Pathol..

[40] Rolnik D.L., da Silva Costa F., Lee T.J., Schmid M., McLennan A.C. (2018). Association between fetal fraction on cell-free DNA testing and first trimester markers for pre-eclampsia. Ultrasound Obstet. Gynecol..

[41] Bauer M., Hutterer G., Eder M., Majer S., Leshane E., Johnson K.L., Peter I., Bianchi D.W., Pertl B. (2006). A prospective analysis of cell-free fetal DNA concentration in maternal plasma as an indicator for adverse pregnancy outcome. Prenat. Diagn..

[42] Poon L.C., Musci T., Song K., Syngelaki A., Nicolaides K.H. (2013). Maternal plasma cell-free fetal and maternal DNA at 11–13 weeks’ gestation: Relation to fetal and maternal characteristics and pregnancy outcomes. Fetal Diagn Ther..

[43] Rolnik D.L., O’Gorman N., Fiolna M., van den Boom D., Nicolaides K.H., Poon L.C. (2015). Maternal plasma cell-free DNA in the prediction of preeclampsia. Ultrasound Obstet. Gynecol..

[44] Lau T.W., Leung T.N., Chan L.Y.S., Lau T.K., Chan A.K.C., Tam W.H., Lo Y.M.D. (2002). Fetal DNA clearance from maternal plasma is impaired in preeclampsia. Clin. Chem..

[45] Scgeffer P., Wirjosoekarto S., Becking E., Weiss M., Bax C., Oepkes D., Sistermans E., Henneman L., Bekker M. (2021). Association between low fetal fraction in cell-free DNA testing and adverse pregnancy outcome: A systematic review. Prenat. Diagn..

[46] Becking E.C., Wirjosoekarto S.A., Scheffer P.G., Huiskes J.V., Remmelink M.J., Sistermans E.A., Bax C.J., Weiss J.M., Henneman L., Bekker M.N. (2021). Low fetal fraction in cell-free DNA testing is associated with adverse pregnancy outcome: Analysis of a subcohort of the TRIDENT-2 study. Prenat. Diagn..

[47] Zaki-Dizaji M., Shafiee A., Kohandel Gargari O., Fathi H., Heidary Z. (2023). Maternal and fetal factors affecting cell-free fetal DNA (cffDNA) fraction: A systematic review. J. Reprod. Infertil..

[48] Lee T.J., Rolnik D.L., Menezes M.A., McLennan A.C., da Silva Costa F. (2018). Cell-free fetal DNA testing in singleton IVF conceptions. Hum. Reprod..

[49] Lee M.S., Cantonwine D., Little S.E., McElrath T.F., Parry S.I., Lim K.H., Wilkins-Haug L.E. (2015). Angiogenic markers in pregnancies conceived through in vitro fertilization. Am. J. Obstet. Gynecol..

[50] Morano D., Rossi S., Lapucci C., Pittalis M.C., Farina A. (2018). Cell-free DNA fetal fraction in early- and late-onset fetal growth restriction. Mol. Diagn. Ther..

[51] Jakobsen T.R., Clausen F.B., Rode L., Dziegiel M.H., Tabor A. (2012). High levels of fetal DNA are associated with increased risk of spontaneous preterm delivery. Prenat. Diagn..

[52] Quezada M.S., Francisco C., Dumitrascu-Biris C., Nicolaides K.H., Poon L.C. (2015). Fetal fraction of cell-free DNA in maternal plasma in the prediction of spontaneous preterm delivery. Ultrasound Obstst. Gynecol..

[53] Luo Y., Xu L., Ma Y., Yan X., Hou R., Huang Y., Liao X., Liu Y., Wang D., Jiang L. (2023). Association between the first and second-trimester cfDNA fetal fraction and spontaneous preterm birth. Expert Rev. Mol. Diagn..

